# Erratum: On the differentiation between trait and state food craving: Half-year retest-reliability of the *Food Cravings Questionnaire-Trait-reduced* (FCQ-T-r) and the *Food Cravings Questionnaire-State* (FCQ-S)

**DOI:** 10.1186/s40337-014-0033-z

**Published:** 2014-11-28

**Authors:** Adrian Meule, Carina Beck Teran, Jasmin Berker, Tilman Gründel, Martina Mayerhofer, Petra Platte

**Affiliations:** Institute of Psychology, University of Würzburg, Würzburg, Germany; Hospital for Child and Adolescent Psychiatry, LWL University Hospital of the Ruhr University Bochum, Heithofer Allee 64, 59071 Hamm, Germany

After publication of this work [1], we noted that we inadvertently missed to acknowledge that this publication was funded by the German Research Foundation (DFG) and the University of Würzburg in the funding program Open Access Publishing.
